# Altered fecal microbial and metabolic profile reveals potential mechanisms underlying iron deficiency anemia in pregnant women in China

**DOI:** 10.17305/bjbms.2022.7091

**Published:** 2022-07-08

**Authors:** Haixia Chen, Weigang Wu, Shuming Tang, Rong Fu, Xia Gong, Hu Hou, Junfa Xu

**Affiliations:** 1 Department of Clinical Laboratory, Shenzhen People’s Hospital (The Second Clinical Medical College of Jinan University; The First Affiliated Hospital of Southern University of Science and Technology), Shenzhen, Guangdong, China; 2 Department of Infectious Disease, Shenzhen People’s Hospital (The Second Clinical Medical College of Jinan University; The First Affiliated Hospital of Southern University of Science and Technology), Shenzhen, Guangdong, China; 3 Department of Obstetrics, Shenzhen People’s Hospital (The Second Clinical Medical College of Jinan University; The First Affiliated Hospital of Southern University of Science and Technology), Shenzhen, Guangdong, China; 4 Department of Clinical Immunology, Institute of Clinical Laboratory Medicine, Guangdong Provincial Key Laboratory of Medical Molecular Diagnostics, Guangdong Medical University, Dongguan, Guangdong, China

**Keywords:** Iron deficiency anemia, pregnant women, microbiome, metagenomics, metabolic profiling, streptococcus, catechol

## Abstract

The gut microbiome and its metabolism may provide crucial insight into the cause of iron deficiency anemia (IDA) in pregnant women. This study aimed to investigate the effect of the gut microbiome and its related metabolites on pregnant women with iron deficiency (ID) and IDA. Maternal cubital venous blood and stool samples were collected from healthy control pregnant women (HC, non-anemic, n = 10), pregnant women with ID non-anemia (ID, n = 10), and IDA (n = 10). All groups were subjected to fecal metagenomics and metabolomics. The composition and function of the gut microbiome were, then, compared in pregnant women with ID and IDA with HC after excluding the possibility of inflammation and insufficient iron absorption capacity. Whole-genome shotgun libraries were prepared by quantifying metagenomic DNA samples with Quant-iT PicoGreen dsDNA Assay. The levels of 41 microbial species, including 21 Streptococci and ten metabolites (catechol), which could serve as siderophores, were increased. In contrast, three Bacteroides and six metabolites were decreased in pregnant women with IDA (*p* < 0.05). The Kyoto Encyclopedia of Genes and Genomes (KEGG) pathway analysis indicated that the bio-pathways, including biosynthesis of siderophore group non-ribosomal peptides (*p* < 0.01), ATP-binding cassette transporters (*p* < 0.05), and membrane transport of the gut microbiota (*p* < 0.01) in IDA patients were expressed differently compared with HC. Correlation analysis also indicates that these increased bacteria formed strong co-occurring relationships with metabolites in the occurrence and development of IDA in pregnant women. The present study identified that streptococci and catechol (fecal metabolite) were significantly increased in pregnant women with IDA. Therefore, adjusting the intestinal homeostasis using long-term living and eating habits on oral Streptococcus in pregnant women with IDA before iron supplementation may be more conducive to iron supplementation, thus providing novel therapies for IDA.

## INTRODUCTION

The World Health Organization (WHO) defines anemia during pregnancy as hemoglobin (Hb) <11 g/dL at any time during pregnancy, including mild (10–10.9 g/dL), moderate (7.0 and 9.9 g/dL), and severe anemia (<7.0 g/dL) [1]. Anemia during pregnancy harms pregnant women and fetuses, for example, low birth weight [2], preterm birth, and small-for-gestational-age live birth [3]; severe anemia during pregnancy even leads to preeclampsia [4] and maternal mortality/morbidity [5].

Anemia is the most common nutritional disorder affecting a quarter of the world’s population, for which the most common cause during pregnancy is iron deficiency (ID) [6]. Iron deficiency anemia (IDA) is that the synthesis of Hb and iron-containing enzymes is reduced due to lack of iron, for which the main causes during pregnancy are insufficient iron content in the diet [7], iron absorption disorder (e.g., iron absorption disability and dietary iron predation of gut microbiome) [7], increased iron requirement/uptake of the placenta and fetus [7,8], and increased need to fuel red blood cell mass [9]. Once the stored iron is exhausted, it is difficult to replenish through food alone, so iron supplementation is indispensable. For instance, the commonly used oral irons include polysaccharide iron complex, ferrous fumarate, ferrous succinate, ferrous sulfate, ferrous gluconate, and iron protein succinate. Other forms of iron are obtained through injection, such as iron sucrose and iron dextran [10]. Nevertheless, oral iron retains intolerance or unsatisfactory efficacy, high-concentration of iron damages the intestinal mucosa and inhibits beneficial bacteria [11]. Moreover, compliance issues such as vomiting and gastrointestinal reaction make it less effective as only a limited amount of iron can be absorbed from the gut [12]; injection iron exerts limitations due to individual differences, for example, allergies [13].

Adults need 20 mg iron/day to support erythropoiesis (80%) and other cellular needs (20%) [14]. Cells obtain iron by displaying transferrin receptor 1 on the cell membrane, which binds iron-containing transferrin and then internalizes the complex into endosomes [15]. Chronic inflammation from infections or other causes limits iron availability and contributes to anemia; in addition, high iron level (>100 mg elemental iron/day) increases hepcidin expression which decreases iron adsorption by inhibiting iron transporter expression [16].

Regarding bacteria, they compete with body for iron by secreting siderophores, for example, catecholate, phenolate, hydroxamate, and carboxylate, in a low-iron environment [17]. Siderophores, which are a chemically diverse type of secondary metabolites [18], identify organic ligands that exhibit specificity for iron. They acquire iron by specific uptake systems and are regulated according to iron availability [19]. For Gram-positive bacteria, which have no outer membrane, siderophores enter directly by ATP binding cassette (ABC) transporter. Whereas for Gram-negative bacteria, since they have an outer membrane, the b barrel receptor on the outer membrane helps recognizing siderophores [17].

For pregnant women, the need for iron is more urgent compared to other adults [20]. As iron is pivotal for blood cell mass and blood volume, IDA, or ID increase the risk of infant morbidity and mortality [20].

The present study aimed to deep analyze and identify the reason for ID and IDA in pregnant women. The participants have similar iron requirement and dietary iron intake. These data would provide soundly scientific evidences for the clinical treatment of ID and IDA in pregnant women.

## MATERIALS AND METHODS

### Cohorts

Healthy control pregnant women (HC, n = 10, pregnancy time 168.5 ± 38.70 day), and pregnant women with ID non-anemia (ID, n = 10, pregnancy time 172.9 ± 36.16 day), and IDA (n = 10, pregnancy time 188.6 ± 28.45 day) were enrolled in this study. All of the women partaking in this study signed an informed consent before enrollment. All the pregnant women underwent prenatal examination in Shenzhen People’s Hospital from May 2019 to June 2020. When the subjects were included in the study, we had conducted a dietary assessment on the subjects and selected pregnant women with a balanced diet, excluding vegetarians, inflammation, liver disease, kidney disease, dyslipidemia, thalassemia, and pregnant women with iron supplement in recent 3 months. Inclusion criteria are follows: (1) singleton pregnancy and (2) pregnant women during the second and third trimesters (not include the first trimester with the following two reasons: (1) in Shenzhen, pregnant women generally do health care in obstetrics after 13 weeks, so there are relatively few patients in early pregnancy; (2) the increase of blood volume in early pregnancy is small, while in middle and late pregnancy is large. To balance the factor of hemodilution caused by the increase of blood volume, the pregnant women in the middle and late pregnancy are studied. There were pregnant women during the second and third trimesters in each group, and the range of gestational stages is exhibited in Table 1. The pregnant women with ID were diagnosed according to the WHO (Hb <11 g/dL) [1], and those with IDA were defined according to the WHO (serum ferritin <15 mcg/L) and not presented infection [21]. No participants have taken antibiotics or probiotics during pregnancy. This study was approved by the Ethics Committee of Shenzhen People’s Hospital (Ethics No. LL-KY-2019424).

**TABLE 1:**
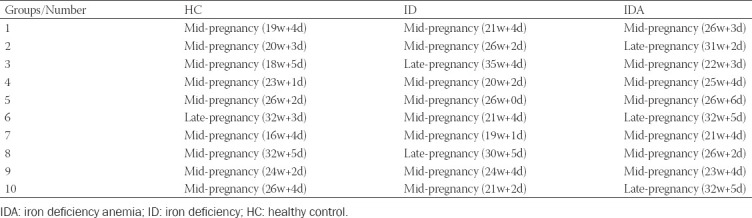
Gestational week (week+day) in each group

### Sample handling

Maternal cubital venous blood (5 mL) was collected on an empty stomach in the morning. An automatic biochemical analyzer (AU5800, Beckman Coulter Laboratory System Corp., Suzhou, China) was used to detect serum iron, C-reactive protein (CRP), alanine aminotransferase (ALT), aspartate aminotransferase (AST), and total bile acid (TBA) level. Hb, interleukin-6 (IL-6), serum ferritin (SF), and serum hepcidin were detected by Sysmex automatic blood analyzer (XN-9000, Sysmex Medical Electronics Corp., Shanghai, China), automatic chemiluminescence immunoassay analyzer (E 601, Roche Diagnostics Corp., Shanghai, China), Automatic chemiluminescence immunoassay analyzer (UniCel DxI 800, Beckman Coulter Laboratory System Corp., Suzhou, China), and manual enzyme immunoassay (Wuhan Merck Biotechnology Corp., China), respectively.

Feces were collected by the same protocol as at hospital or home, which were delivered immediately at low temperatures and frozen at −80°C until metabolomic profiling or DNA extraction.

### Metabolite profiling from stool samples

Each 100 mg stool sample was homogenized in 400 μL water by a bead mill (TissueLyser II, QIAGEN), the aqueous homogenates were aliquoted for UHPLC-QE Orbitrap/MS analysis. The UHPLC system (1290, Agilent Technologies) with a UPLC HSS T3 column (2.1 mm*100 mm, 1.7 μm) coupled to Q Exactive Orbitrap (Thermo Fisher Scientific) were used. The formic acid (0.1%) and ammonium acetate (5 mM) were solvent A for positive (ES+) and negative (ES-), respectively, while acetonitrile was Solvent B. About 2 μL fecal sample was added at 4°C for analysis. The gradient elution of solvent B was as follows: 1%, 0-1 min; 99%, 8 min; 99%, 10 min; 1%, 10 min; and 1%, 12 min. The spray voltage was 3.8 kV for ES+, and 3.1 kV for ES−, with the capillary temperature of 320°C. About 70-1000 mass-to-charge ration (m/z) masses were acquired. The resolved power of full MS and MS/MS was set to 70000 and 17500, respectively.

The raw data were converted into mzXML format by ProteoWizard and preprocessed with R package XCMS v3.1.3 – including peak identification, peak filtration, and peak alignment. The processed data included m/z, retention time, and peak intensity. Impurity peaks and duplicate identifications were eliminated. For each data set, we removed the compounds that were present in <50% of samples within a study. The identification of the tentative metabolite was mapped in MS/MS database by standards and HMDB database.

Orthogonal partial least squares discriminant analysis was performed to evaluate the differences in metabolic profiles among HC, ID, and IDA. Normalization ensured that the median and quartiles of metabolite content were close to the same level. The analysis was performed by R package MetaboAnalyst. All the observed and predicated compounds were imported in KEGG database. The metabolites with variable important in projection ≥1, *p* < 0.05, and >1.25-fold changes were further analyzed.

The QC-RFSC algorithm of the R language StatTarget package was used to correct the signal peak of characteristic in each sample (metabolite), and the correction effect of each metabolite was recorded. During the signal data acquisition process, QC samples (a mixture of all samples at equal amounts) were inserted at the beginning, end, and middle positions to record the signal drift; if there was no signal drift, the signal strength of QC samples remained unchanged. After correcting the signal drift, the correction effect was proved to be good if QC sample points were clustered together in the PCA chart.

### DNA extraction and metagenomic sequencing

QIAamp DNA Stool Mini Kit (QIAGEN) was used to extract stool DNA. Whole-genome shotgun libraries were prepared by quantifying metagenomic DNA samples with Quant-iT PicoGreen dsDNA Assay (Thermo Fisher Scientific) and normalized to 50 pg/μL. Illumina sequencing libraries were prepared from DNA (100-250 pg) by Nextera XT DNA library Preparation Kit (Illumina). Batches of 24, 48, or 96 libraries were pooled by transferring equal volumes of each library using an Echo 550 Liquid Handler (Labcyte). Insert sizes and concentrations for each pooled library were determined, libraries were sequenced on the HiSeq 2500 platform (Illumina), targeting ~6 Gb of sequence/sample with 101 base pair, paired-end reads.

Sequencing system runs RTA3, an implementation of Real-Time Analysis software, on the instrument Compute Engine. RTA3 extracts intensities from images received from the camera, performs base calling, assigns a quality score to base calls, aligns to PhiX, and reports data in InterOp files for viewing in Sequencing Analysis Viewer. Sequencing System uses two-channel sequencing, which requires only two images to encode the data for four kinds of DNA bases, one from the red channel, and one from the green channel. The intensity of each cluster is extracted from the red and green images and compared with each other, producing four distinct populations. Each population corresponds to one base. The base calling process determines, in which population does each cluster belongs to. No calls occur when a cluster fails to pass filter (PF), registration fails, or shifted off the image. A no call is identified as N.

During the run, RTA3 filters raw data, deletes reads that do not meet the data quality threshold, and removes overlapping and low-quality clusters.

For two-channel analysis, RTA3 uses a population-based system to determine the chastity (intensity purity measurement) of a base call. Clusters PF when no more than one base call in the first 25 cycles has a chastity below a fixed threshold. PhiX alignment is performed at cycle 26 on a subset of tiles for clusters that passed filter. Clusters that do not PF are not base called and not aligned.

A quality score (Q-score) is a prediction of the probability of an incorrect base call. A higher Q-score implies that a base call is higher quality and more likely to be correct. After the Q-score is determined, results are recorded in base call files. The Q-score succinctly communicates small error probabilities. Quality scores are represented as Q (X), where X is the score. Q (X) shows the relationship between a quality score and error probability as follows: Q10 indicates error probability of 0.1 (1 in 10), Q20 indicates error probability of 0.01 (1 in 100), Q30 indicates error probability of 0.001 (1 in 1000), and Q40 indicates error probability of 0.0001 (1 in 10,000). Our selection criteria were those for X >20.

### Read-level quality control and metagenomic profiling

Raw sequencing reads were analyzed by KneadData version 0.5.1 for quality control (based on Trimmomatic) and de-hosting (based on Bowtie2). Before and after KneadData, FastQC will be used to test the rationality and effect of quality control. Kraken2 was used to identify the species contained in the samples, and then, Bracken was used to predict the actual relative abundance of the species in the samples. Species with relative abundance <0.1% in at least five samples were excluded from the study. Sequence-based metagenomic species annotation methods were more comprehensive and accurate than assembly-based species annotation. Starting with quality control and removing the reads of the host gene, functional profiling was performed using HUMAnN2 version 0.9.4 in UniRef90 mode. HUMAnN2 initially maps metagenomic readings to the pan-genomes of species identified during taxonomic profiling. The coding sequences in these pan-genomes have been pre-annotated into their respective UniRef90 families. Reads that are not aligned with a pan-genome were mapped to UniRef90 for translating search with DIAMOND. Per-sample gene abundances were sum-normalized to ppm units. Based on species abundance and functional abundance, and abundance cluster analysis, Principal Coordinates Analysis (PCoA) and non-metric multidimensional scaling analysis (NMDS) dimensionality reduction analysis (species only) and sample cluster analysis could be performed. When grouping information was available, LEfSe biomarker mining analysis and metabolic pathway comparative analysis could be performed to discover the differences in species composition and functional composition between samples.

### Ethical statement

The present study was approved by the Ethics Committee of Shenzhen People’s Hospital (Ethics No. LL-KY-2019424). All pregnant women signed an informed consent before enrollment.

### Statistical analysis

Data were analyzed by GraphPad, and presented as mean ± SD. Data of levels of biochemical indexes were analyzed by One-way-ANOVA. Differentially abundant metabolites (332) and microbial species (1939) were clustered to identify similar trend of iron in ID and IDA compared with HC. Multiomic correlation between metabolites and microbial features was applied to speculate the mechanistic associations.

## RESULTS

### Physiological and biochemical indexes changed in pregnant women with ID and IDA

**FIGURE 1:**
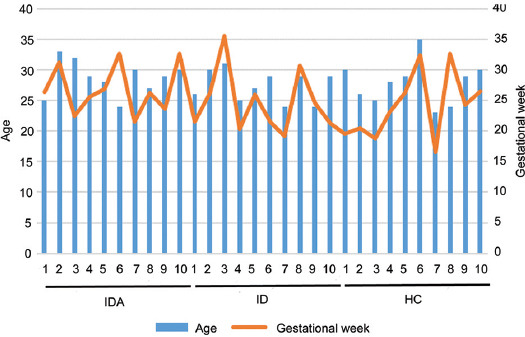
The levels of age and gestational week in HC, ID, and IDA. IDA: iron deficiency anemia; ID: iron deficiency; HC: healthy control.

**FIGURE 2:**
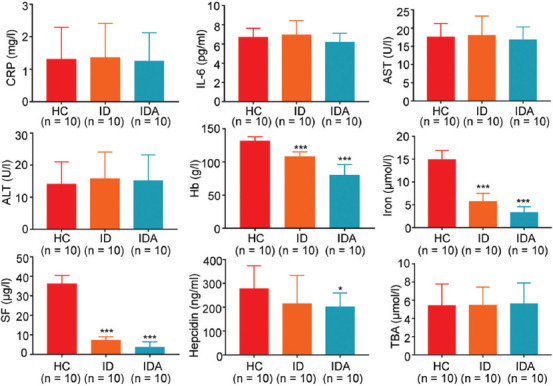
The levels of CRP, IL-6, AST, ALT, TBA, Iron, Hb, SF, and hepcidin in HC, ID, and IDA. The values are shown as mean ± SD.*p < 0.05, **p < 0.01, ***p < 0.001 versus HC. CRP: C-reactive protein; IL-6: Interleukin 6; AST: alanine aminotransferase; ALT: Aspartate aminotransferase; TBA: Total bile acid; Hb: hemoglobin; SF: serum ferritin; DA: iron deficiency anemia; ID: iron deficiency; HC: healthy control.

No significant difference in age, gestational week (Figure 1 and Table 1), CRP, IL-6, AST, ALT, or TBA was found among HC, ID and IDA; except for Hb (*p* < 0.001), iron (*p* < 0.001), SF (*p* < 0.001), and Hepcidin (*p* < 0.05), which were lower in ID and IDA compared with HC (Figure 2).

Pregnant women were well nourished according to the recorded diet (data not shown), suggesting the sufficiency of dietary iron intake (nutritional status); there was no significant difference in iron requirements (gestational weeks) among the pregnant women. Therefore, it is critical to discover the cause of ID and IDA.

Chronic inflammation limits iron availability and contributes to anemia [16]. Iron, which is an essential metal for cell survival, is regulated by hepcidin. Increased serum hepcidin concentrations preclude the flow and efflux of extra- and intra-cellular iron [22]. Herein, compared to HC, IL-6 exerted no significant change in ID or IDA, eliminating the effect of inflammation on available iron in blood, in while hepcidin exhibited significant decrease in IDA, indicating the normality of iron absorption capacity and the insufficiency of iron in intestine of pregnant women. Consequently, we turned our attention to the gut microbiome and its related metabolites.

### Disturbed microbial composition and function in gut of pregnant women with ID and IDA

**FIGURE 3:**
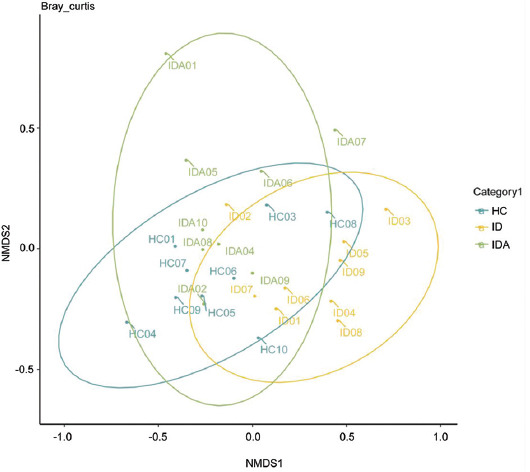
PCA analyses of variation between the bacterial communities presenting in all biopsy samples based on Bray-Curtis distance matrices. Each data point represents an individual sample. IDA: iron deficiency anemia; ID: iron deficiency; HC: healthy control.

To explore whether gut microbiota changed, we performed fecal metagenome. A mean of 21 million clean reads in each sample were obtained after the qualification of raw data. A PCoA was performed to investigate the extent of similarity of the microbial communities in HC, ID, and IDA based on NMDS. A notable separation was observed between HC and IDA (Figure 3), suggesting that the microbiota composition of IDA was significantly different from that of HC.

**FIGURE 4:**
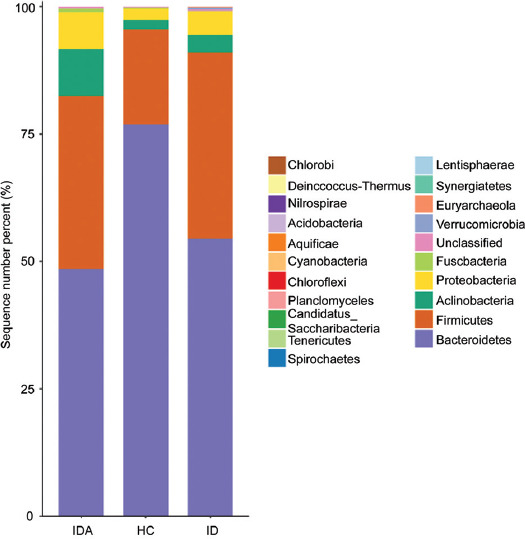
Relative abundance of the main phyla of the intestinal microbiota in HC, ID, and IDA. IDA: iron deficiency anemia; ID: iron deficiency; HC: healthy control.

At the phylum level, ID and IDA were characterized by a significantly higher Firmicutes/Bacteroidetes ratio than HC (Figure 4).

**FIGURE 5:**
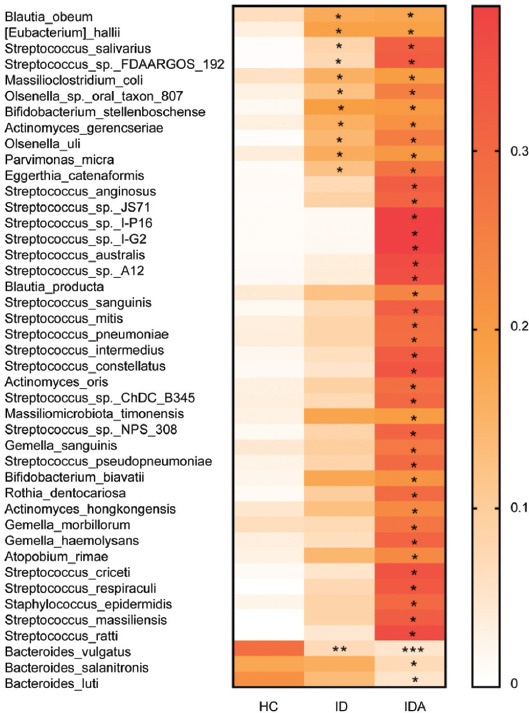
The shift of gut microbiota in HC, ID, and IDA of pregnant women according to the metagenomic data, only variables showing significant changes in IDA/ID related to controls were used to generate the heat map. *p < 0.05, **p < 0.01, ***p < 0.001 versus HC. IDA: iron deficiency anemia; ID: iron deficiency; HC: healthy control

Afterward, the species level distribution and species level abundance of intestinal flora for HC, ID, and IDA were illustrated (Figure 5, Tables 2 and 3). As shown in Table 2, compared to HC, *Bacteroides vulgatus* (*p* < 0.01, *p* < 0.001) was decreased and 11 species were increased (*p* < 0.05) in species level of both ID and IDA. Moreover, *Bacteroides luti* (*p* < 0.05) and *Bacteroides salanitronis* (*p* < 0.05) were decreased, and 29 species were increased specifically in species level of IDA (*p* < 0.05).

**TABLE 2:**
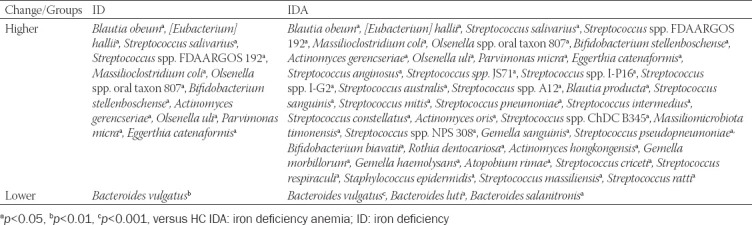
Species level distribution shifts for ID and IDA

**TABLE 3:**
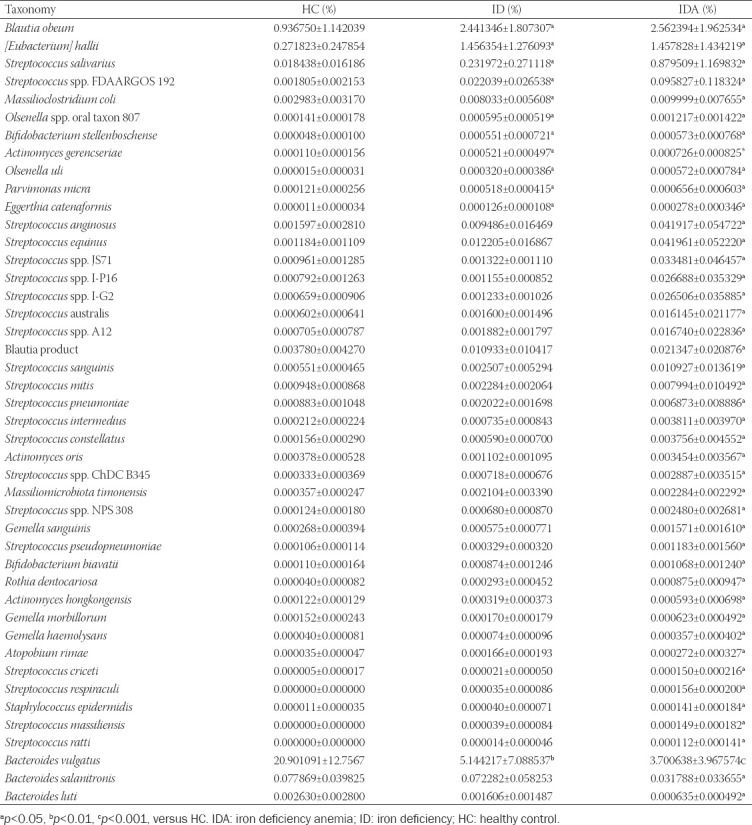
Species level abundance of intestinal flora in HC and pregnant women with ID and IDA

As shown in Table 3, compared to HC, the *B. vulgatus* (*p* < 0.01, *p* < 0.001) also decreased and 11 species were increased (*p* < 0.05) in both ID and IDA; moreover, *B. luti* (*p* < 0.05) and *B. salanitronis* (*p* < 0.05) were decreased, and 30 species were increased specifically in species level abundance of IDA (*p* < 0.05).

**FIGURE 6:**
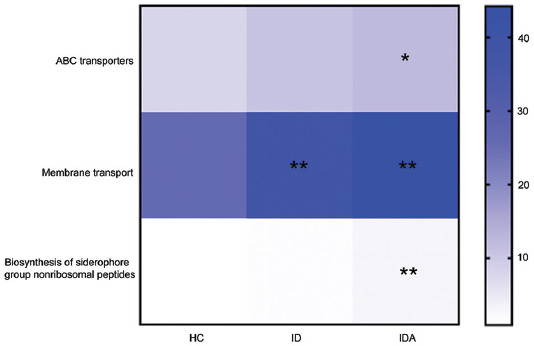
KEGG pathway reconstructions reveal functions that are enriched or underrepresented of gut microbiota in HC, ID and IDA, only variables showing significant changes in IDA/ID related to controls were used to generate the heat map. *p < 0.05, **p < 0.01, ***p < 0.001 versus. HC. IDA: iron deficiency anemia; ID: iron deficiency; HC: healthy control.

As to functional analysis, there were three main pathways enriched in ID and IDA, including ABC transporters (*p* < 0.05), membrane transport (*p* < 0.01), and biosynthesis of siderophore group non-ribosomal peptides (*p* < 0.01) (Figure 6).

Iron is an essential nutrient in most living bacteria, for which the common way to acquire iron is to secrete metabolites (e.g., siderophores); in which, ABC transporters involved in the uptake of siderophores are commonly used [23]. Hence, metabolic profiling is essential to further figure out how gut microbiota function iron predation in ID and IDA.

### Broad metabolic shifts in pregnant women with ID and IDA

**FIGURE 7:**
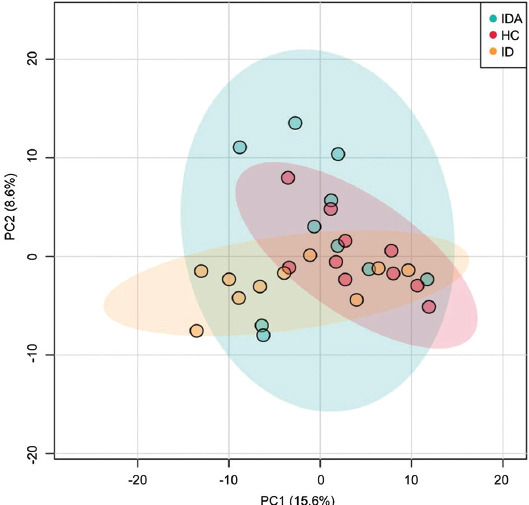
PCA analytical of fecal metabolites results from HC, ID, and IDA. Each data point represents an individual sample. IDA: iron deficiency anemia; ID: iron deficiency; HC: healthy control.

Subsequently, compared with HC, the fecal metabolic shifts in ID and IDA were analyzed by UHPLC-QE Orbitrap/MS analysis. In total, 332 metabolites matched against unique standards were measured for differential abundance analysis, and the metabolomics profiles of HC, ID, and IDA were separated by PCA analysis (Figure 7).

**FIGURE 8:**
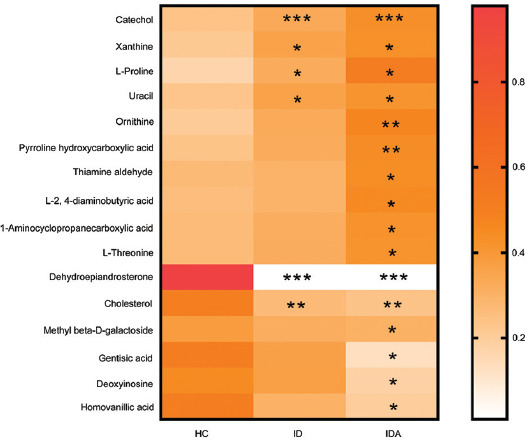
The shift of fecal metabolites in HC, ID and IDA of pregnant women according to the metabolic profiling, only variables showing significant changes in IDA/ID related to controls were used to generate the heat map. *p < 0.05, **p < 0.01, ***p < 0.001 versus HC. IDA: iron deficiency anemia; ID: iron deficiency; HC: healthy control.

Compared with HC, 16 biomarkers were significantly changed in ID and IDA, the levels of catechol (*p* < 0.001), xanthine (*p* < 0.05), uracil (*p* < 0.05), and L-proline (*p* < 0.05) were significantly increased, while the levels of dehydroepiandrosterone (DHEA) (*p* < 0.001) and cholesterol (*p* < 0.01) were significantly decreased in both ID and IDA; moreover, the levels of the remaining ten biomarkers (*p* < 0.05, *p* < 0.01) were specifically and significantly changed in IDA (Figure 8 and Table 4).

**TABLE 4:**
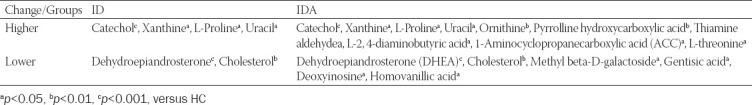
Metabolic shifts in pregnant women with ID and IDA

### Correlation network analysis of altered gut microbiota and fecal metabolites

**FIGURE 9:**
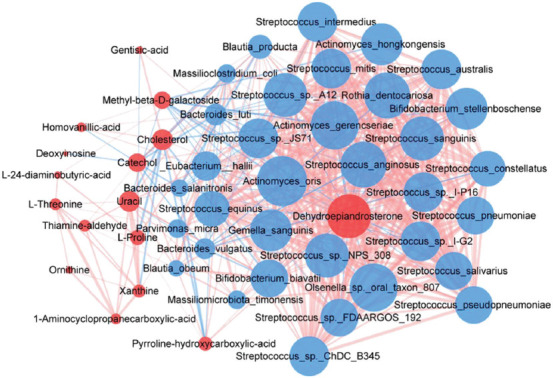
The correlation network between gut microbiota and its metabolites. Red and blue dots indicate the metabolites and bacteria, respectively. The area of the dot represents the content or proportion. The larger the dot, the higher the content or proportion. Edges between nodes indicate Spearman’s negative (light blue) or positive (light red) correlation, respectively. The thickness of the line represents the interaction intensity. The larger the action intensity is, the thicker the line is.

A network was constructed based on the Spearman correlation analysis to explore potential reciprocal interactions between altered gut microbiota and metabolites. The increased metabolites such as catechol and uracil were positively correlated with several Streptococci, and negatively correlated with Bacteroides, while the decreased metabolites such as DHEA were on the contrary (Figure 9).

## DISCUSSION

As far as we are aware, this is the first study that studies the changes of the gut microbiome and metabolome in pregnant women with IDA by an integrated multiomic framework, indicating that IDA in pregnant women is related to not only iron intake, but also microbial traits (e.g., the enrichment of oral Streptococcus, and the secreted siderophores).

Reduced serum iron is associated with increased IL-6 and hepcidin [24]; therefore, less iron is absorbed on account of the reduction of intestinal ferroprotein by hepcidin. However, this study discovered that IL-6 was not significantly changed in ID or IDA, eliminating the effect of inflammation on available iron in blood, while serum hepcidin was significantly decreased in IDA, eliminating the abnormality of the iron absorption capacity in pregnant women. Consequently, we turned our attention to the gut microbiome and its related metabolites.

The impact of IDA on gut microbiota has been evaluated in infants and children [25]. The previous study indicated that distinct gut microbiota signatures were observed in IDA patients compared with HC [25]. This study, further, investigated the gut microbiota pattern in pregnant women with ID and IDA using the same technique (next-generation sequencing). We also observed that ID and IDA displayed obvious alteration in gut microbiota composition. IDA exhibited higher relative abundance of 41 bacteria, most of which belong to Streptococci. This is different compared with data acquired from infants and children, which indicated that abundance of *Enterobacteriaceae*, *Veillonellaceae*, and *Coriobacteriaceae* were altered in IDA group [25]. Biosynthesis pathways including ABC transporters, membrane transport, and siderophore group non-ribosomal peptides were enhanced in IDA. Iron is essential for most living bacteria, for which a common way to acquire iron is to secrete metabolites (e.g., siderophores); in which ABC transporters involved in the uptake of siderophores are commonly used [23]; additionally, iron is essential for numerous bacterial physiological processes including DNA replication and transcription, while pathogenic bacteria have developed complicated strategies to gain iron from host sources [26]. Hence, metabolic profiling is essential for us to figure out how gut microbiota function in ID and IDA.

Metabolites are intuitive manifestations of function variation in gut microbiota, directly or indirectly affecting the normal physiological functions of the body. A recent study performed metabolite profiling to analyze the impact of ID on juvenile monkeys using their urine and plasma. Alteration of maltose and microbial-derived metabolites was observed in ID [27]. Herein, 332 metabolites matched against unique standards were measured, and the metabolomics profiles of HC, ID, and IDA were separated. The amount of many metabolites was changed in ID and IDA compared with HC. After eliminating the effect of inflammation and iron absorption disability on available iron in blood, the plundering of iron by intestinal flora was concerned. We inferred the possible mechanism of iron predation by bacteria based on changes in the gut microbiome, and the analysis of non-targeted metabolomics was to further verify our speculation.

In addition to changes in ID-induced metabolites, siderophores and their precursors were found. Compared with HC, 16 biomarkers were changed in ID and IDA, among which, six biomarkers including catechol, xanthine, uracil, L-proline, DHEA, and cholesterol were changed in ID and IDA. Ten biomarkers were specifically changed in IDA. Among them, nine metabolites are closely related to iron utilization and iron predation. For instance, catechol was a metabolite which exerted the most significant upregulation in ID and IDA, consistent with the result of metagenomics; while catecholate, which exhibited the most significant downregulation in ID and IDA, was a common type of siderophore mainly produced under ID [16], suggesting that the competition of bacteria for iron is a major cause of IDA, and the ABC transport system in bacteria is overactivated for absorbing siderophores. Ferritin is an iron-storage protein and ferritin iron can be directly chelated by microbial siderophores, the reduction of Fe^3+^ to Fe^2+^ in ferritin iron mobilization can be mediated by xanthine oxidoreductase with xanthine [28], the increased xanthine in ID and IDA indicated that xanthine either releases iron from ferritin for usage in body or benefits iron predation of bacteria, but it was difficult to judge which siderophore is stronger. Proline hydroxylases are an Fe^2+^-dependent enzyme [29], which explained the increase of L-proline in ID; L-proline is also related to prokaryotic-type ABC transporters which are associated with iron transport [30]; therefore, the results in metabolomics may not only be the cause of bacterial iron predation, but also the result of ID, which needed to be distinguished. Uracil DNA glycosylases, which possesses Fe-S cluster and can be inactivated by Fe^2+^, can excise uracil from DNA to avoid mutation under stress [31], consistent with the increased change of uracil under ID. Ornithine is a precursor for siderophore production, and supplementation with ornithine greatly enhanced siderophore production [32], consistent with the increased change of ornithine in IDA. Siderophores are mainly absorbed by ABC transporters on the bacterial membrane, the same as thiamine; therefore, the upregulated absorption rate of ABC transporters might be responsible for the increased thiamine aldehyde in IDA. L-2,4-diaminobutyric acid is a potential precursor of siderophores [33]. The conversion of 1-aminocyclopropanecarboxylic acid (ACC) to ethylene is mediated by ACC oxidase (an iron-dependent enzyme) [34], explaining the elevation of ACC in IDA. L-threonine is a part of acinetobactin siderophores [35], explaining the elevation of L-threonine in IDA. The remaining seven metabolites, including pyrroline hydroxycarboxylic acid, DHEA, cholesterol, methyl beta-D-galactoside, gentisic acid, deoxyinosine, and homovanillic acid (HVA), needed to be further studied and might be new mechanisms of iron predation. Among which, pyrroline hydroxycarboxylic acid may be a new possible chelating agent for iron [36]; however, the exact mechanism between it and ABC transporters needed to be further explored. However, it is been also described that the microbiome might compete with the host for iron resources when the mineral is limited [37]. Thus, IDA might be caused for an unknown cause and that shortage might induce microbial changes toward the production of siderophores.

Finally, we found that the increased metabolites, which mainly included catechol and uracil, were positively correlated with several Streptococci, while negatively correlated with Bacteroides. Although there was no significant difference in blood cholesterol level among HC, ID, and IDA, the fecal cholesterol in ID and IDA was significantly reduced, consistent with the phenomenon of microorganism in assimilating cholesterol and producing siderophores [38]. Gentiolic acid is an endogenous siderophore produced during co-evolution with bacteria [39], the reason for its decline in IDA remains unclear. However, the decreased metabolites such as DHEA were on the contrary. DHEA resists iron-induced oxidative stress and apoptosis, suggesting that DHEA may be positively correlated with iron levels in response to iron-related effects [40]. Methyl beta-D-galactoside and deoxyinosine are related to phosphate and amino acid transporters (one type of ABC transporters). In neurotransmitter disturbance, iron is positively associated with increased HVA [40], which may fill in new evidence for the brain gut axis theory.

The metagenomics and metabolomic data in ID resemble those in IDA but with less dramatic changes, suggesting that intestinal dysbiosis and microbial metabolic alterations are gradual during the instauration of anemia. Therefore, it’d be useful in clinical management (early diagnosis of ID) can prevent the development of IDA by modifying the diet (microbiome).

## CONCLUSIONS

Collectively, the present study identified metabolic changes in the microbial community associated with IDA in pregnant women at a certain time. Streptococcus (the key bacteria), and catechol (fecal metabolite), which could lead to the development of IDA in pregnant women, were significantly increased. The observed changes in the gut microbiome and metabolic profile are a cause or consequence of anemia cannot be known. Therefore, the effect of long-term living and eating habits on microbial, especially the oral Streptococcus in pregnant women with IDA, can be further analyzed. This could provide novel therapies for IDA such as adjusting the intestinal homeostasis and limiting the secretion of siderophores.

At present, there are several limitations within this study. Relatively small sample size with limited clinical data, which will be enlarged to further verify the conclusions. ID, fecal microbial, and metabolic profile altered in ID at the same time, it is hard to figure out which came first through these changes were confirmed to further worsen ID. Further, mechanism involved in oral Streptococcus and its secreted siderophores remains unknown. Lack of causal relationships. Finally, a correlation analysis between continuous traits (e.g., Hb and ferritin) and the microbiome composition or metals is not performed.
